# Culturing captures members of the soil rare biosphere

**DOI:** 10.1111/j.1462-2920.2012.02817.x

**Published:** 2012-09

**Authors:** Ashley Shade, Clifford S Hogan, Amy K Klimowicz, Matthew Linske, Patricia S McManus, Jo Handelsman

**Affiliations:** 1 Department of Molecular, Cellular and Developmental Biology, Yale UniversityKline Biology Tower, 219 Prospect St, New Haven, CT 06520, USA; 2 Department of Plant Pathology, University of Wisconsin-Madison1630 Linden Drive, Madison, WI 53706, USA; 3 Department of Bacteriology, University of Wisconsin-MadisonMicrobial Sciences Building, 1550 Linden Drive, Madison, WI 53706, USA

## Abstract

The ecological significance of rare microorganisms within microbial communities remains an important, unanswered question. Microorganisms of extremely low abundance (the ‘rare biosphere’) are believed to be largely inaccessible and unknown. To understand the structure of complex environmental microbial communities, including the representation of rare and prevalent community members, we coupled traditional cultivation with pyrosequencing. We compared cultured and uncultured bacterial members of the same agricultural soil, including eight locations within one apple orchard and four time points. Our analysis revealed that soil bacteria captured by culturing were in very low abundance or absent in the culture-independent community, demonstrating unexpected accessibility of the rare biosphere by culturing.

Microorganisms are the most abundant organisms on Earth and represent an unfathomably high level of diversity (Whitman *et al*., 1998; Rappé and Giovannoni, 2003; Schloss and Handelsman, 2007). Traditional culturing techniques expose a small subset of this vast environmental microbial diversity (Lane *et al*., 1985; Hugenholtz and Pace, 1996; Rondon *et al*., 2000). To reveal greater diversity, culturing was replaced first by Sanger sequencing of 16S rRNA genes (Olsen *et al*., 1986; Rappé and Giovannoni, 2003) and then by high-throughput sequencing (Liu *et al*., 2007). High-throughput sequencing revealed that detection of microorganisms of extremely low abundance was inadequate by either sequencing method (Sogin *et al*., 2006).

Microorganisms of extremely low abundance have been designated ‘the rare biosphere’ (Sogin *et al*., 2006). The ecological significance of rare microorganisms is just beginning to be understood (Pedrós-Alió, 2012). One hypothesis is that rare members represent a dormant seed bank. Members of this seed bank may become active at random (Epstein, 2009), or in direct response to changes in the environment, for instance, to initiate community recovery after disturbance (Epstein, 2009; Lennon and Jones, 2011). This hypothesis is supported by a recent investigation of Baltic Sea bacterioplankton responses to organic carbon additions, wherein rare members increased in abundance from less than 10 sequences to as many as thousands after carbon amendment (Sjöstedt *et al*., 2012). Similarly, a study in the Western English Channel showed that community members in low abundance were persistent over time, and that, in a few cases, populations of rare members occasionally bloomed (Caporaso *et al*., 2011). However, there also are situations in which rare members are hypothesized to be less important for the community, such as when populations are becoming extinct or are between favourable environments (Pedrós-Alió, 2012). Despite advances in sequencing depth that reveal rare organisms and a few intriguing observations using these sequencing technologies, the significance of the rare biosphere remains obscure (Hugenholtz and Pace, 1996; Sogin *et al*., 2006; Huse *et al*., 2010). However, because members of the rare biosphere may provide novel products and processes, bioprospecting for these organisms has been made a priority (Reid and Buckley, 2011).

Soil harbours a complex microbial consortium that contains a preponderance of low-abundance microorganisms (e.g. Roesch *et al*., 2007), representing perhaps the most significant habitat of the rare biosphere. To estimate the contribution of rare members to soil microbial communities, a clone library of 16S rRNA genes, including 13 000nearly full-length sequences, was analysed (Elshahed *et al*., 2008). The results suggested that many rare members represented either phylogenetically divergent or generally uncommon lineages for the soil environment (Elshahed *et al*., 2008). The goal of the present study was to understand the contributions of cultured and as-yet-uncultured members of soil bacterial communities to community structure. To directly compare culture-based and culture-independent communities, it was important to have both assessed using the same technology. Thus, we compared taxa identified by pyrosequencing of the 16S rRNA genes isolated by culture-based and culture-independent methods.

Soil samples from eight locations within one apple orchard were collected twice per year over 2 years. DNA was extracted directly from soil on the day of collection (culture-independent), and from bacteria cultured from soil on rhizosphere isolation medium (RIM) modified by replacing nystatin with cycloheximide (100 µg ml^−1^) to inhibit fungal growth (Buyer, 1995). Plates were incubated for 6 days at room temperature. Pyrosequencing of the 16S rRNA gene V3-V4 variable region was performed with primers 515/806, and sequences were analysed using default QIIME v. 1.2.1 workflow (see Supporting information, Caporaso *et al*., 2010). Pyrosequencing generated 83 992 quality sequences across 58 samples, representing 5822 operational taxonomic units (OTUs) containing at least 97% sequence identity (Table S1, Fig. 1A). These sequences are available (MG-RAST ID 4487654.3). To ensure a conservative estimate of richness, singleton OTUs (i.e. OTUs observed once throughout the entire dataset) were omitted (Fig. 1B). There were no global differences in the composition of bacterial communities across locations in the orchard (*P* = 0.87, 0.89) or time (*P* = 0.33, 0.21), as assessed by analysis of similarity from ranked Bray–Curtis distances and from Morista–Horn similarity respectively.

**Fig. 1:**
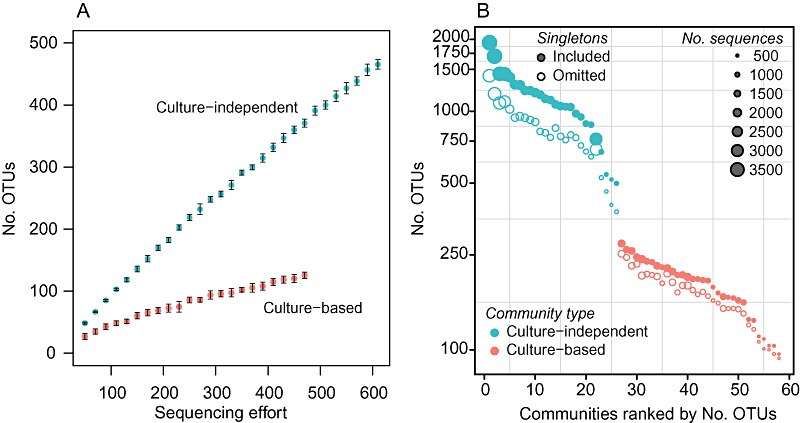
Community structure varies between culture-based and culture-independent assessments. A. To determine the completeness of the sequencing effort, rarefaction was performed from a minimum of 50 sequences to the median number of sequences observed across all samples (610 sequences, as per QIIME default parameters for the script alpha_rarefaction.py). Error bars are standard error around the mean of 10 subsamples at each level of sequencing effort. B. Omitting singletons has minimal effect on community structure. Communities are ranked by number of OTUs. Closed symbols represent communities including singleton OTUs, and open symbols represent communities without singleton OTUs. Thus, the difference on the *y*-axis between the closed and open symbols depicts the change in the number of OTUs within a community after omitting singletons. Symbols are scaled in size to the total number of sequences observed within a community before rarefaction.

Most OTUs in the collection of cultured microorganisms (61%) were not detected in the communities described by culture-independent means (Fig. 2A). We identified the most abundant OTUs as those positioned before the beginning of the ‘tail’ of the log-scale rank abundance curve (Fig. S1), as a way of partitioning abundant taxa from rare taxa in a community. For the top 26 OTUs, we observed a range of 300 to 7102 total sequences. Of the 26 most-prevalent OTUs across the entire dataset, 22 were abundant only among the cultured organisms and were in low abundance or below detection among the culture-independent sequences (Fig. 2B). The four that were prevalent in both groups were OTUs 4789, 6271, 27 and 5980. Moreover, 23 of 26 dominant OTUs among the cultured members were rare among the uncultured members (Fig. S2A), and 23 of 26 dominant OTUs among the uncultured were rare among the cultured organisms (Fig. S2B). These observations indicate that most OTUs obtained by culturing under standard conditions (see Appendix S1) were rare among community members represented by pyrosequencing DNA directly isolated from soil. These cultured rare members likely represent a fraction of the total rare biosphere, but a subset that would have been undiscovered by pyrosequencing at the given sequencing depth (a depth suitable for uncovering patterns of microbial beta diversity, e.g. Kuczynski *et al*., 2010). Thus, a combination of culture-dependent and culture-independent analysis described the soil microbial community more comprehensively than either approach alone and uncovered members of the rare biosphere.

**Fig. 2:**
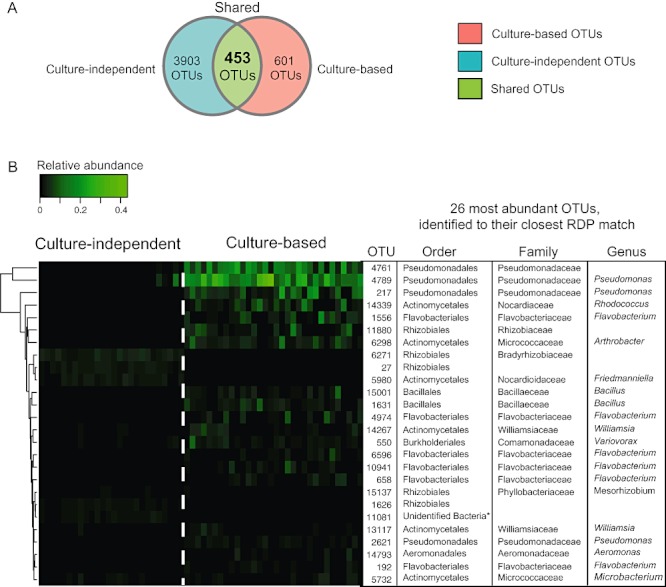
Culture-based and culture-independent analyses of soil bacterial communities. A. Number of OTUs shared between culture-based and culture-independent analyses, and number of OTUs unique to each analysis. B. Heat map of 26 most abundant OTUs in the dataset, organized by response patterns (clusters shown by dendrogram). Columns are community samples from the culture-independent method (*n* = 26, left side of the dashed white line) or the cultured-based method (*n* = 32, right side of the dashed white line). Each OTU is in a row and colour intensity indicates its relative abundance, with brighter green indicating higher abundance. Asterisk indicates that the OTU could not be identified to the Order level.

Although culture-based analysis of microbial communities has been largely replaced by sequence-based studies such as 16S rRNA gene analysis and metagenomics, our study demonstrates an advantage of including culturing in characterization of the microbial communities. Additionally, cultivation provides access to diverse characteristics of each microorganism, offering a rich platform for physiological analyses, which could advance bioprospecting, one of the motivations for pursuing the rare biosphere (Reid and Buckley, 2011). However, not all rare community members will be captured readily by culturing. Culturing from the murine gut, for example, revealed many abundant members of the microbial community (Goodman *et al*., 2011), and thus in that habitat, deep sequencing of culture-independent samples will likely provide better access to rare members. Therefore, success of cultivating rare members will depend, first, on the life strategies of microorganisms inhabiting a niche (e.g. copiotrophic or oligotrophic, Dethlefsen and Schmidt, 2007; Fierer *et al*., 2007) and, second, on the precise conditions of cultivation.

Life strategies of microorganisms are important for understanding microbial community ecology (Dethlefsen and Schmidt, 2007; Fierer *et al*., 2007), and perhaps also for understanding the roles of rare members in the community. Cultivation is thought to select for opportunistic ‘weed’ species of bacteria, or copiotrophs, that grow quickly in resource-rich conditions common to many culture media (e.g. Garland *et al*., 2001). Some members of the soil rare biosphere may be copiotrophs waiting for favourable resources to flourish. This mechanism for maintenance of copiotrophic rare taxa would be in agreement with the ‘storage effect’ (Warner and Chesson, 1985), which posits that strategies of temporal bet-hedging (e.g. dormancy) promote persistence of populations.

Furthermore, some soil bacterial phyla are associated with copiotrophic or oligotrophic life strategies. For example, in one study, soil *Acidobacteria* had a negative relationship with carbon concentration and were classified as oligotrophs, while *Betaproteobacteria* and *Bacteroidetes* had a positive relationship with carbon concentration and were classified as copiotrophs (Fierer *et al*., 2007). Although not all phyla could be assigned to a copiotrophic or oligotrophic life strategy, the authors observed that *Acidobacteria* were prevalent in communities assessed using culture-independent techniques but underrepresented in isolate collections, whereas *Betaproteobacteria* and *Bacteroidetes* were common in isolate collections (Fierer *et al*., 2007). Similarly, in our soil analysis we detected no *Acidobacteria* in the culture-based collection, but detected many in the culture-independent collection (Fig. S3). By proportion, *Bacteroidetes*-affiliated taxa comprised most of the culture-based members, and were also prevalent among culture-independent OTUs. There was a similar pattern for *Betaproteobacteria*.

The precise conditions of cultivation, including choice of medium, also determine which community members are detected using culture-based methods. Our results show that even standard cultivation conditions can uncover members of the rare biospehere. We used a modified RIM, a medium developed specifically to inhibit growth of *Bacillus mycoides*, a bacterium with a filamentous colony morphology that obscures smaller bacterial colonies. Although RIM lacks five common amino acids, it supported growth of a similar number of bacterial colonies and more bacterial genera than a medium containing Casamino Acids (Buyer, 1995). Furthermore, we identified a similar number of bacterial taxa on RIM and another medium, 0.1× tryptic soy agar, which is commonly used for isolation of soil bacteria (Fig. S4). Both media revealed taxa representing four phyla (*Actinobacteria*, *Bacteroidetes*, *Firmicutes* and *Proteobacteria*) and three *Proteobacteria* classes (*Alpha*-, *Beta*- and *Gammaproteobacteria*; Fig. S4). Thus, while RIM is a selective medium, it does not appear to inhibit soil bacteria other than *B. mycoides*.

Members of the rare biosphere of soil were accessible using standard cultivation conditions, suggesting that we may not be as ignorant about the soil rare biosphere as previously suspected. Our study also supports the prediction that members of the rare biosphere may mediate functions that are essential for community viability, potentially acting as keystone species. For example, readily cultured soil microorganisms that were in low abundance based on culture-independent pyrosequencing, such as some *Rhizobiales* taxa and *Streptomyces* (e.g. Janssen, 2006), are extremely important in nitrogen fixation and production of secondary metabolites respectively, which are critical functions in soil ecology. As microorganisms in low abundance will likely prove important in other habitats, understanding the rare biosphere is therefore a key feature of global microbial ecology. The results presented here show that organisms known to play important ecological roles are members of the rare biosphere, that combining culture-based methods with next generation sequencing can provide a richer portrait of microbial diversity than does either approach alone, and that the diminishing use of culturing in modern microbiology may obscure critical dimensions of community structure and function.

In summary, our findings demonstrate that through culturing of soil samples, microbiologists have been studying members of the rare biosphere intensively since the 19th Century. Moreover, the ecological importance of many readily cultured members of the soil community is well established, validating that members of the rare biosphere contribute vital functions to the soil community.

## Supporting information

**Figure S1.** Rank abundance distribution of OTUs from singleton-omitted, rarefied dataset from soil.

**Figure S2.** Heatmap visualizations of the 26 most abundant OTUs within the (A) culture-based and (B)culture-independent communities, and organized within each map by similar response patterns. Columns are communities, and rows are OTUs. Please note differences in scale of relative abundances for A and B. *Variovorax* (OTU 550) was the only abundant OTU shared across both cultured-based and culture-independent communities. OTUs marked with an asterisk could not be resolved to the Order level.

**Figure S3.** Frequency of OTUs across Bacteria phyla and *Proteobacteria* class for (A) culture-independent and (B) culture-based communities.

**Figure S4.** Comparison of taxa detected in rhizosphere isolation medium (RIM) and 0.1× tryptic soy agar (TSA) at the (A) phylum level and (B) *Proteobacteria* class. (C) Venn analysis of OTUs detected on each type of medium. OTUassignments were made with Ribosomal Database Project assignment of full-length 16S RNA gene sequences from clone libraries of metagenomic DNA extracted from a pool of isolates for each culturing media. RIM was ultimately selected for cultivation because it inhibited the growth of filamentous *Bacillus mycoides*, which often obscured quantification of smaller, non-filamentous colonies on TSA.

**Table S1.** Summary of sequencing effort and number of OTUs detected in culture-based, culture-independent, and the total dataset. Note that the total is not necessarily the sum of the culture-based and culture-independent (e.g. some OTUs were shared across both communities).
