# Supplementary material for: Culturing captures members of the soil rare biosphere
Source: Environ Microbiol. 2012 Sep;14(9):2247–52. doi: 10.1111/j.1462-2920.2012.02817.x (PMC3466410; doi:10.1111/j.1462-2920.2012.02817.x)
Supplement: Supplementary file 1 [file emi0014-2247-SD1.pdf]

Supplemental Online Materials

for

Culturing captures members of the soil rare biosphere

A Shade, CS Hogan, AK Klimowicz, M Linske, PS McManus, and J Handelsman

Supplemental Methods

The study site was an apple orchard in southern Wisconsin (42°58'N, 89°28'W). Soil cores 10 cm in diameter and 5 cm deep were collected under the north and south sides of eight trees at four time points: 08 and 27 May 2008, and 01 and 22 May 2009. Each was homogenized in its collection bag.

Five-gram soil subsamples were prepared for plating as described previously (Gilbert *et al.*, 1996). Briefly, 5 g of soil were diluted with 25 ml of sterile H_2O, shaken for 1 minute, and then sonicated for 30 seconds in a bath sonicator. Soil was allowed to settle, and then 1/10 dilutions were made. For each core, we plated duplicate series of 100 µl of 10^{-3}, 10^{-4}, and 10^{-5} dilutions on rhizosphere isolation medium (Buyer, 1995) modified by replacing nystatin with cycloheximide (100 µg/ml) to inhibit fungal growth. Plates were incubated at ambient temperature for 6 days before colonies were scraped, and then the plates diluted from north and south cores were pooled within a site (either 8, 10, or 12 plates pooled total per sample, as the 10^{-3} dilution was often overgrown and excluded). Next, each pool of colonies was homogenized by mixing with a spatula and vortexing. The pooled samples were stored in 10% glycerol at -80º C prior to DNA extraction with GenElute Bacterial Genomic DNA kit (Sigma-

Aldrich, St. Louis, MO) protocol for Gram-positive bacteria. For the culture-independent description of bacterial communities, genomic DNA was isolated directly from the soil within 6 hours of collection using the Power Soil kit (MoBio, Carlsbad, CA) with published modifications (Cadillo-Quiroz *et al.*, 2006).

Barcoded-16S gene amplification was performed on 64 samples (8 sites x 4 time points x 2 approaches, culture-based and culture-independent) by the University of Colorado-Boulder pyrosequencing lab, and submitted for pyrosequencing on a 454 Life Sciences Genome Sequencer FLX (Roche Diagnostics, Branford, CT) at Eugencore, South Carolina, using prokaryotic 16S rRNA gene primers 515/806 to target the V3-V4 variable regions. Of 64 total samples, we failed to obtain sequence from six samples, which were omitted from analysis. Sequence quality control was performed using the QIIME v 1.2 default parameters, which included a minimum quality score of 25, a minimum sequence length of 200, a maximum length of 1000, and no ambiguous bases permitted, and no primer mismatches (Caporaso *et al.*, 2010b). Operational taxonomic units (OTUs) were assigned based on 97% sequence similarity using uclust (Edgar, 2010), and the most abundant sequence within each OTU was used as the representative for alignment and taxonomic assignment. Alignments were performed using PyNAST (Caporaso *et al.*, 2010a) and assignments made using the RDP Classifier (Wang *et al.*, 2007) with 0.8 minimum confidence and 0.001 minimum e-values.

There were no global differences in the composition of bacterial communities across sites or time points, as assessed by analysis of similarity of ranked Bray-Curtis distances in the Primer-e V6 software ($p = 0.87$ and 0.33). Heatmap and Venn analyses were performed in the R environment for statistical computing (R Development Core Team).

To assess the reproducibility of community structure across GenElute and Power Soil DNA extraction methods, we built clone libraries containing 16S rRNA genes amplified from two samples, for a total of four libraries (two samples and two extraction methods each). 16S rRNA genes were amplified from soil metagenomic DNA using primers 27f and 1492r pooled from 4-6 separate 50-µL PCR reactions and purified using the Promega Wizard kit.

Purified PCR products were then dialyzed against dH_20, on membrane filters (Millipore VSWP02500) for 1 hour at room temperature. We ligated approximately 90 ng insert DNA into 50 µg of pGEM T easy vector (Promega) overnight at 4° C. Following ligation, 1 µl of the ligation reactions was mixed with 50 µl of electro-competent *E. coli* DH5α cells, incubated on ice for 20 min, transferred to a 1-mm electroporation cuvette, and pulsed at 2500 V, 4.8 – 5.8-ms time constant. The cells recovered in SOC media for 1 hour at 37 ^{o}C with shaking before plating on LB medium amended with 50 µg/mL ampicillin plus ChromoMax^{TM} IPTG (isopropyl thiogalactoside)/ X-GAL (5-bromo-4-chloro-3-indolyl-beta-D-galactopyranoside) for blue/white colony selection (Fisher). After overnight growth, white colonies were picked with toothpicks and patched on LB medium amended with ampicillin and grown overnight. Cells were transferred to 96-well plates and pelleted by centrifuging at 14,000 G for 15 minutes. The culture medium was discarded and replaced with 100 µl sterile water. The plate was heated to 95° C for 10 min to lyse the cells. One µl of the cell suspension was applied to the PCR reaction using primers M13f and 357r. BigDye sequencing reactions were performed on successful amplifications. Reactions were analyzed by the University of Wisconsin-Madison Biotechnology Center on a 3730 xL DNA Analyzer (Applied Biosystems).

We identified chimeras from aligned sequences with Mallard (Ashelford *et al.*, 2006),

and removed them after confirmation of low quality with BLAST. The remaining analyses were performed in the mothur environment (Schloss *et al.*, 2009). Sequences were aligned against mothur's greengenes reference collection and screened for the same start and end position. After quality control, 417 sequences remained that were used to create a PHYLIP distance matrix (Felsenstein, 1993). This matrix was applied to the neighbor-joining algorithm of clearcut (Evans *et al.*, 2006). We used unweighted UniFrac algorithm (Lozupone and Knight, 2005) to test for differences in structure among groups. We detected no differences between the GenElute and the Power Soil extraction methods (both UniFrac $p > 0.01$). This result was confirmed using the S-Libshuff algorithm (Schloss *et al.*, 2004), applied to the PHYLIP distance matrix (S-Libshuff $p \geq 0.01$).

Supplemental Table

Supplemental Table 1. Summary of sequencing effort and number of OTUs detected in culture-based, culture-independent, and the total dataset. Note that the total is not necessarily the sum of the culture-based and culture-independent (*e.g.,* some OTUs were shared across both communities).

	Culture-based	Culture-independent	Total
No. samples	32	26	58
No. sequences	37645	46347	83992
No. OTUs	1377	4898	5822
No. OTUs, singletons removed	1054	4356	4957
Max. no. sequences per sample	1764	3294	83992
Avg. no. sequences per sample	1176.4	1782.6	1448.1
Min. no. sequences per sample	471	626	471
Max. no. OTUs per sample	253	1410	1410
Avg. no. OTUs per sample	168.8	820.8	461.1
Min no. OTUs per sample	92	379	92
Max no. OTUs per sample, singletons removed	221	1358	1358
Avg. no. OTUs per sample, singletons removed	158.8	800.0	446.2
Min no. OTUs per sample, singletons removed	84	366	84

Supplemental Figures

Supplemental Figure 1

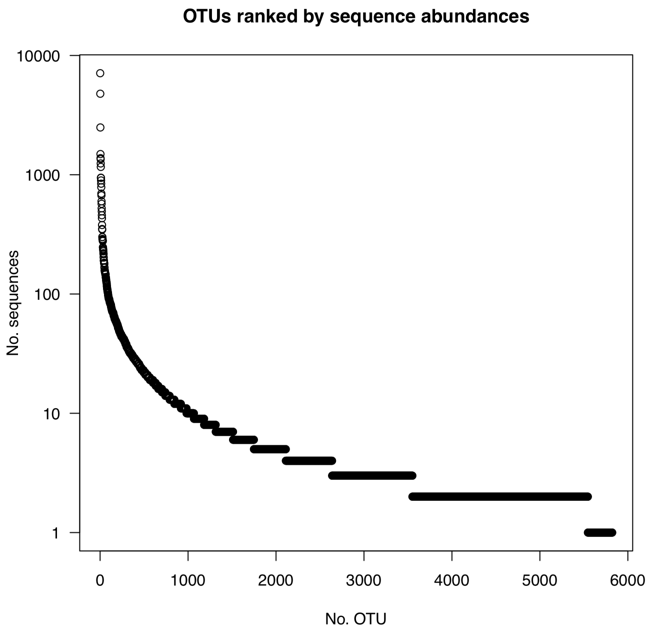

Supplemental Figure 1. Rank abundance distribution of OTUs from singleton-omitted, rarefied dataset from soil.

Supplemental Figure 2.

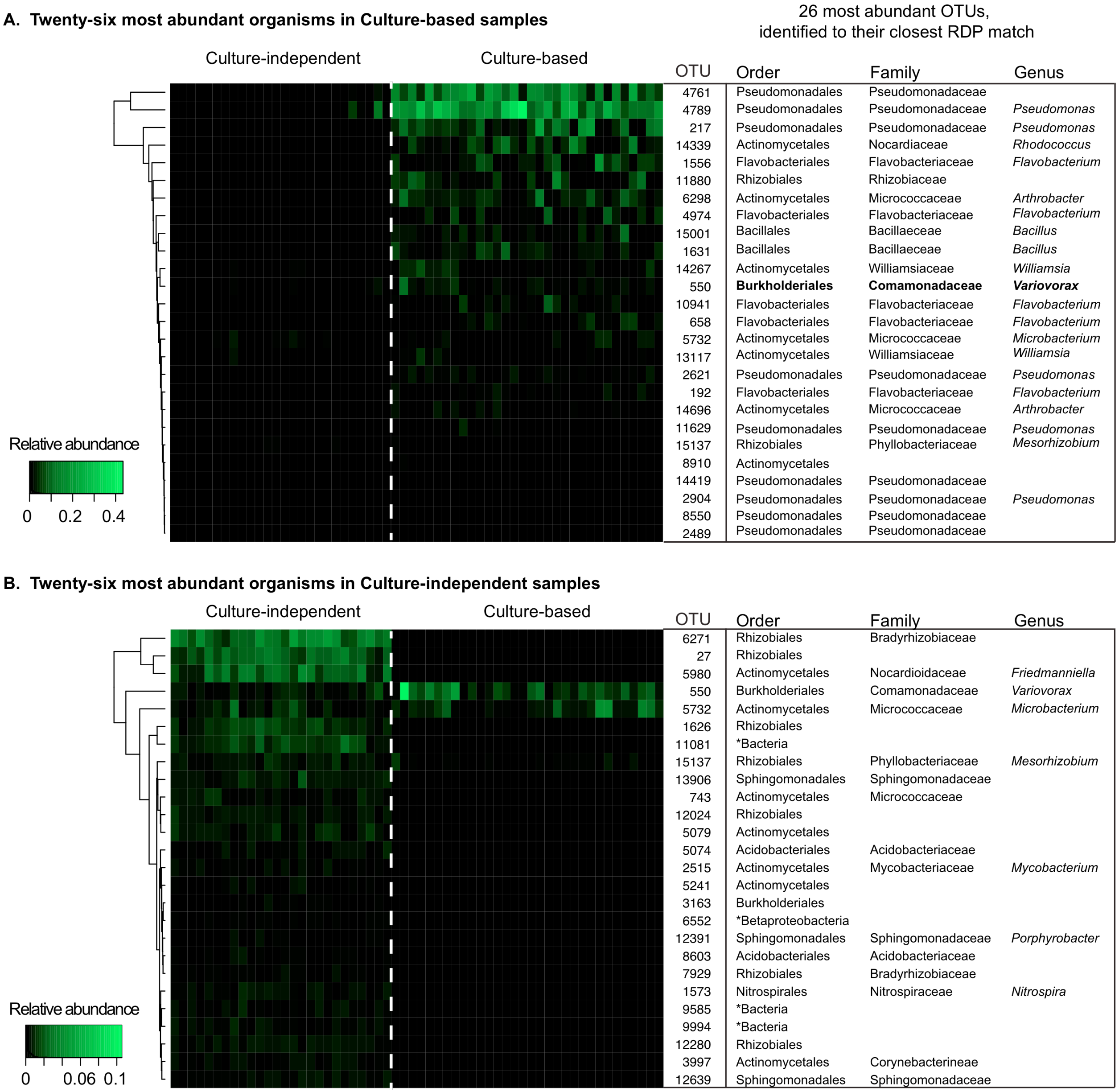

Supplemental Figure 2. Heatmap visualizations of the 26 most abundant OTUs within the (A) culture-based and (B) culture-independent communities, and organized within each map by similar response patterns. Columns are communities, and rows are OTUs. Please note

differences in scale of relative abundances for A and B. *Variovorax* (OTU 550) was the only abundant OTU shared across both cultured-based and culture-independent communities. OTUs marked with an asterisk could not be resolved to the Order level.

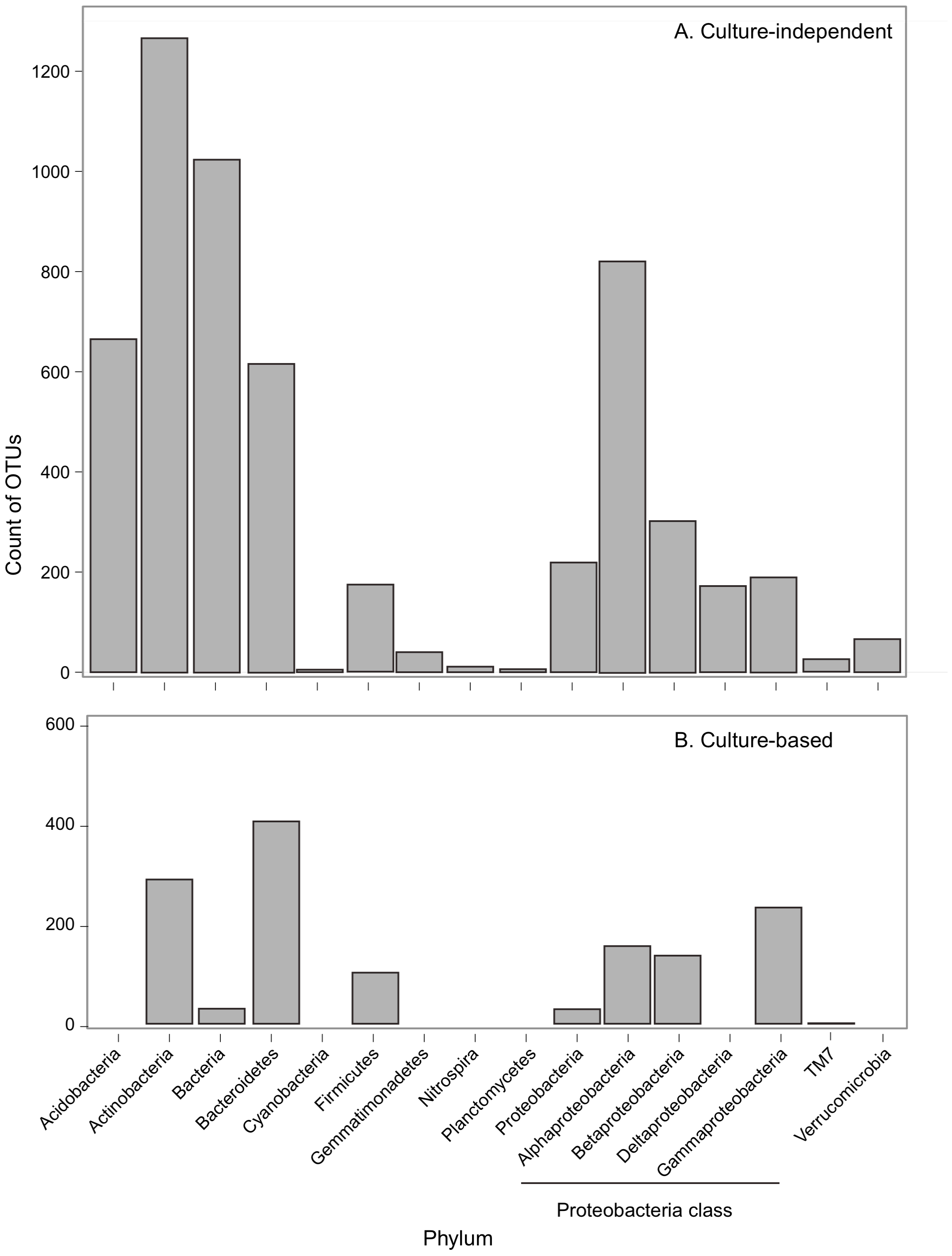

100 **Supplemental Figure 3**. Frequency of OTUs across Bacteria phyla and Proteobacteria class for (a) culture-independent and (b) culture-based communities.

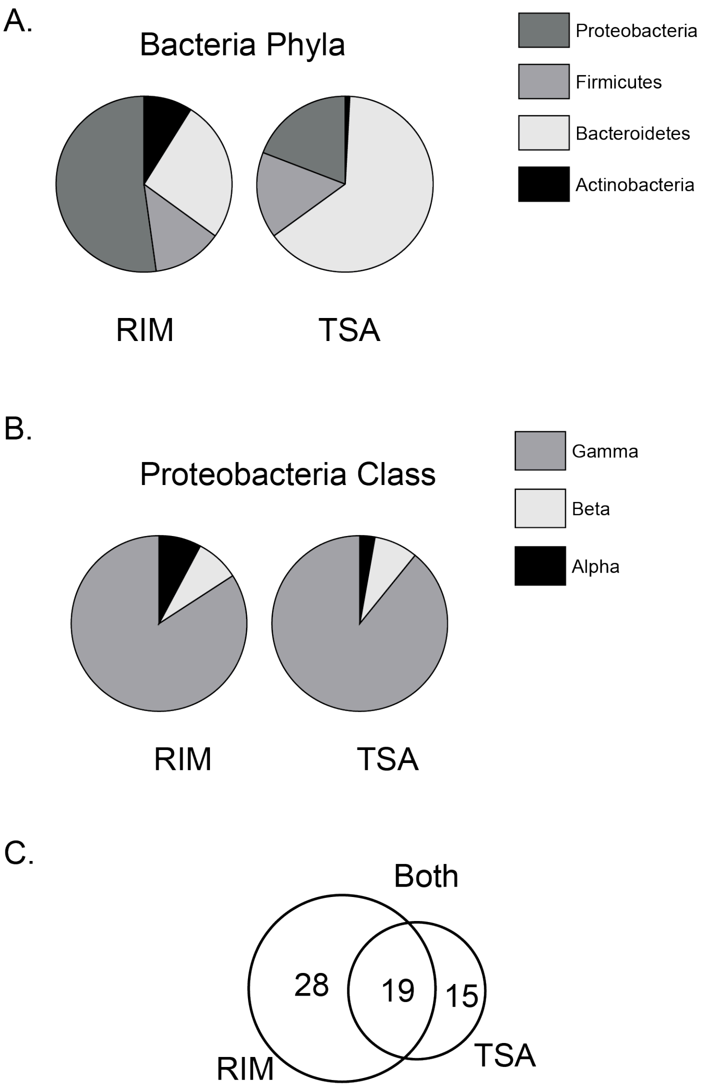

Supplemental Figure 4. Comparison of taxa detected in rhizosphere isolation medium (RIM) and 0.1x tryptic soy agar (TSA) at the (a) phylum level and (b) Proteobacteria class. (c) Venn analysis of OTUs detected on each type of medium. OTU assignments were made with Ribosomal Database Project assignment of full-length 16S RNA gene sequences from clone libraries of metagenomic DNA extracted from a pool of isolates for each culturing media. RIM was ultimately selected for cultivation because it inhibited the growth of filamentous *Bacillus mycoides*, which often obscured quantification of smaller, non-filamentous colonies on TSA.

Supplemental References

Ashelford, K.E., Chuzhanova, N.A., Fry, J.C., Jones, A.J., and Weightman, A.J. (2006) New screening software shows that most recent large 16S rRNA gene clone libraries contain chimeras. *Appl Environ Microbiol* **72**: 5734.

Buyer, J.S. (1995) A soil and rhizosphere microorganism isolation and enumeration medium that inhibits *Bacillus mycoides*. *Appl Environ Microbiol* **61**: 1839-1842.

Cadillo-Quiroz, H., Brauer, S., Yashiro, E., Sun, C., Yavitt, J., and Zinder, S. (2006) Vertical profiles of methanogenesis and methanogens in two contrasting acidic peatlands in central New York State, USA. *Environ Microbiol* **8**: 1428-1440.

Caporaso, J.G., Bittinger, K., Bushman, F.D., DeSantis, T.Z., Andersen, G.L., and Knight, R. (2010a) PyNAST: a flexible tool for aligning sequences to a template alignment. *Bioinformatics* **26**: 266-267.

Caporaso, J.G., Kuczynski, J., Stombaugh, J., Bittinger, K., Bushman, F.D., Costello, E.K. et al. (2010b) QIIME allows analysis of high- throughput community sequencing data. *Nature* **7**: 335-336.

Edgar, R.C. (2010) Search and clustering orders of magnitude faster than BLAST. *Bioinformatics* **26**: 2460-2461.

Evans, J., Sheneman, L., and Foster, J. (2006) Relaxed neighbor joining: a fast distance-based phylogenetic tree construction method. *J Mol Evol* **62**: 785-792.

Felsenstein, J. (1993) Phylip. In *Phylogeny Inference Package version*.

Gilbert, G.S., Clayton, M.K., Handelsman, J., and Parke, J.L. (1996) Use of cluster and discriminant analyses to compare rhizosphere bacterial communities following biological perturbation. *Microb Ecol* **32**: 123-147.

Lozupone, C., and Knight, R. (2005) UniFrac: a new phylogenetic method for comparing microbial communities. *Appl Environ Microbiol* **71**: 8228.

R Development Core Team (2005) R: A language and environment for statistical computing. In. Vienna, Austria: R Foundation for Statistical Computing.

Schloss, P.D., Larget, B.R., and Handelsman, J. (2004) Integration of microbial ecology and statistics: a test to compare gene libraries. *Appl Environ Microbiol* **70**: 5485.

Schloss, P.D., Westcott, S.L., Ryabin, T., Hall, J.R., Hartmann, M., Hollister, E.B. et al. (2009) Introducing mothur: open-source, platform-independent, community-supported software for describing and comparing microbial communities. *Appl Environ Microbiol* **75**: 7537.

Wang, Q., Garrity, G.M., Tiedje, J.M., and Cole, J.R. (2007) Naive Bayesian classifier for rapid assignment of rRNA sequences into the new bacterial taxonomy. *Appl Environ Microbiol* **73**: 5261-5267.
